# Zusammenhänge zwischen freiheitsentziehenden Zwangsmaßnahmen und der Umgebungstemperatur in der stationären psychiatrischen Regelversorgung: Eine retrospektive Datenauswertung

**DOI:** 10.1007/s00115-025-01902-x

**Published:** 2025-09-24

**Authors:** Daniel Kamp, Timo Jendrik Faustmann, Juha Marko Lahnakoski, Claus Lamm, Boryana Todorova, Michaela Jänner, Leonhard Schilbach

**Affiliations:** 1 Klinik und Poliklinik für Psychiatrie und Psychotherapie, Medizinische Fakultät, Heinrich-Heine-Universität Düsseldorf, Düsseldorf, Deutschland; 2 Abteilung für Allgemeine Psychiatrie 2, LVR-Klinikum Düsseldorf, Düsseldorf, Deutschland; 3 Klinik für Psychiatrie und Psychotherapie, Ludwig-Maximilians-Universität München, München, Deutschland; 4 Abteilung für Biometrie, LVR-Klinikum Düsseldorf, Düsseldorf, Deutschland; 5 Institut für Psychologie der Kognition, Emotion und Methoden, Fakultät für Psychologie, Universität Wien, Wien, Österreich

## Hintergrund

Temperatureinflüsse auf den menschlichen Körper, insbesondere klimatische Extremereignisse wie beispielsweise Hitzewellen, stellen ein erhöhtes Risiko für die Verschlechterung der psychischen Gesundheit dar [1]. Insbesondere rasche Temperaturanstiege auf über 30 °C gelten hierbei als kritisch und sind mit einem Anstieg aggressiver Verhaltensweisen assoziiert [2]. Hierbei gibt es eine erste Evidenz dafür, dass dieser Zusammenhang sowohl für eine Zunahme notfallmäßiger Vorstellungen psychiatrischer Patient*innen in einer Notaufnahme besteht [3] sowie auch für fremdaggressive Übergriffe in der psychiatrischen Regelversorgung [2].

Der Zusammenhang von Außentemperaturen und der Notwendigkeit zu freiheitsentziehenden Zwangsmaßnahmen (wie Isolationen und Fixierungen, FEZM) sowie seine Beeinflussung durch weitere Determinanten, wie Diagnose, Alter und Geschlecht psychiatrischer Patient*innen, ist jedoch weitestgehend unklar. Aufgrund der Diskussion um mögliche negative Einflüsse durch Temperaturveränderungen gewinnt diese Thematik im Rahmen des sogenannten „menschengemachten Klimawandels“ in Bezug auf die psychische Gesundheit aber weiter an Bedeutung [4].

Ziel der Studie ist es daher, Zusammenhänge zwischen FEZM in einer großen, universitären psychiatrischen Fachklinik (Landschaftsverband Rheinland(LVR)-Klinikum Düsseldorf/Kliniken der Heinrich-Heine-Universität Düsseldorf) zu untersuchen. Vor dem Hintergrund oben genannter Befunde testen wir hierbei die Hypothese, dass besonders heiße Tage mit hohen Außentemperaturen zu einem Anstieg an FEZM führen, obwohl in der Klinik durchgehend und systematisch Anstrengungen zur Verringerung solcher Maßnahmen unternommen werden.

## Methodik

Es erfolgte eine monozentrische Datenauswertung der elektronischen Patient*innenakten der Sommermonate, d. h. Mai bis September der Jahre 2018 bis 2024 der Abteilungen für Erwachsenenpsychiatrie des LVR-Klinikums Düsseldorf. Erhoben wurden die Anzahl der FEZM für jeden Tag als Betrachtungseinheit (*N* = 1071). Es wurde mittels Pearson-Korrelation der Zusammenhang zwischen dem Tagesmaximum der Lufttemperatur des jeweiligen Tages sowie der Anzahl an FEZM untersucht. Hierbei erfolgte die Nutzung der offiziellen Temperaturdaten der Messstation Düsseldorf des Deutschen Wetterdienstes [5], die sich in einem Abstand von 7,6 km zum Klinikum befindet.

Um Effekte für verschiedene Subgruppen abbilden zu können, erfolgten separate Untersuchungen jeweils (1) für Männer und Frauen, (2) für ältere vs. jüngere Patient*innen (Split-half-Aufteilung anhand des medianen Alters der jeweils isolierten bzw. fixierten Patient*innen) und (3) für Fälle mit/ohne Hauptdiagnose einer Erkrankung aus dem schizophrenen Formenkreis.

Separat erfolgte eine Berechnung für die Bereiche > 30 °C, 20–30 °C sowie < 20 °C.

Statistische Berechnungen erfolgten mittels SPSS (Version 28.0.0.0). Das Signifikanzlevel lag hier bei *p* < 0,05 (zweiseitig).

## Ergebnisse

Es wurden 1071 Tage (7 Jahre à 153 Tage) mit 18.726 Patient*innenfällen über die Jahre 2018 bis 2024 ausgewertet. Insgesamt wurden 10.456 männliche (55,8 %), 8262 weibliche (44,1 %) sowie 8 diverse (< 0,1 %) Patient*innenfälle untersucht. Das mittlere Alter lag bei 47,15 Jahren (Spannweite 18–100 Jahre, Standardabweichung (Std-Abw.) 18,05). Insgesamt zeigten sich nach der internationalen statistischen Klassifikation der Krankheiten und verwandter Gesundheitsprobleme, 10. Revision (ICD-10) 1228 (6,6 %) Fälle aus dem Bereich F0 (organische, einschließlich symptomatische psychische Störungen), 5611 (30,0 %) Fälle aus dem Bereich F1 (psychische Verhaltensstörungen durch psychotrope Substanzen), 4432 (23,7 %) Fälle aus dem Bereich F2 (Schizophrenie, schizotype und wahnhafte Störungen), 5574 (29,8 %) Fälle aus dem Bereich F3 (affektive Störungen) sowie 1881 (10,0 %) Fälle aus übrigen Diagnosekategorien nach ICD-10.

Für die Gesamtgruppe zeigte sich eine signifikante positive Korrelation zwischen Tagesmaximum der Lufttemperatur und Anzahl der Fixierungen (*p* < 0,01, r = 0,146) bei insgesamt 1071 Fixierungen, jedoch nicht für die Isolationen.

Für die nach Geschlecht unterteilten Subgruppen zeigten sich lediglich für die Männer signifikante Korrelationen zwischen dem Tagesmaximum der Lufttemperatur und Anzahl der Fixierungen (*p* < 0,01, r = 0,140) sowie für die Isolationen (*p* < 0,01, r = 0,104).

Für die nach Alter unterteilten Subgruppen (jünger als 36 Jahre vs. älter als 36 Jahre) zeigten sich für Fixierungen analog signifikante Korrelationen sowie für die Gesamtkohorte in Bezug auf das Tagesmaximum der Lufttemperatur (*p* = 0,02, r = 0,073 für jünger 36 Jahre sowie *p* < 0,01, r = 0,120 für älter 36 Jahre). Bei den Isolationen zeigte sich hingegen lediglich eine signifikante Korrelation zwischen der Anzahl der Isolationen und dem Tagesmaximum der Lufttemperatur bei Patient*innen über 42 Jahre (*p* < 0,01, r = 0,116).

Für die nach Diagnose unterteilten Subgruppen zeigte sich in Bezug auf Fixierungen eine signifikante Korrelation (*p* < 0,01, r = 0,149) nur für die Subgruppe der Erkrankungen aus dem schizophrenen Formenkreis, aber nicht für die anderen psychiatrischen Hauptdiagnosen nach ICD-10. Dieser Effekt zeigte sich bei den Isolationen nicht. In Bezug auf die Isolationen zeigte sich für keine der Diagnosesubgruppen ein signifikanter Zusammenhang.

**Abb. 1:**
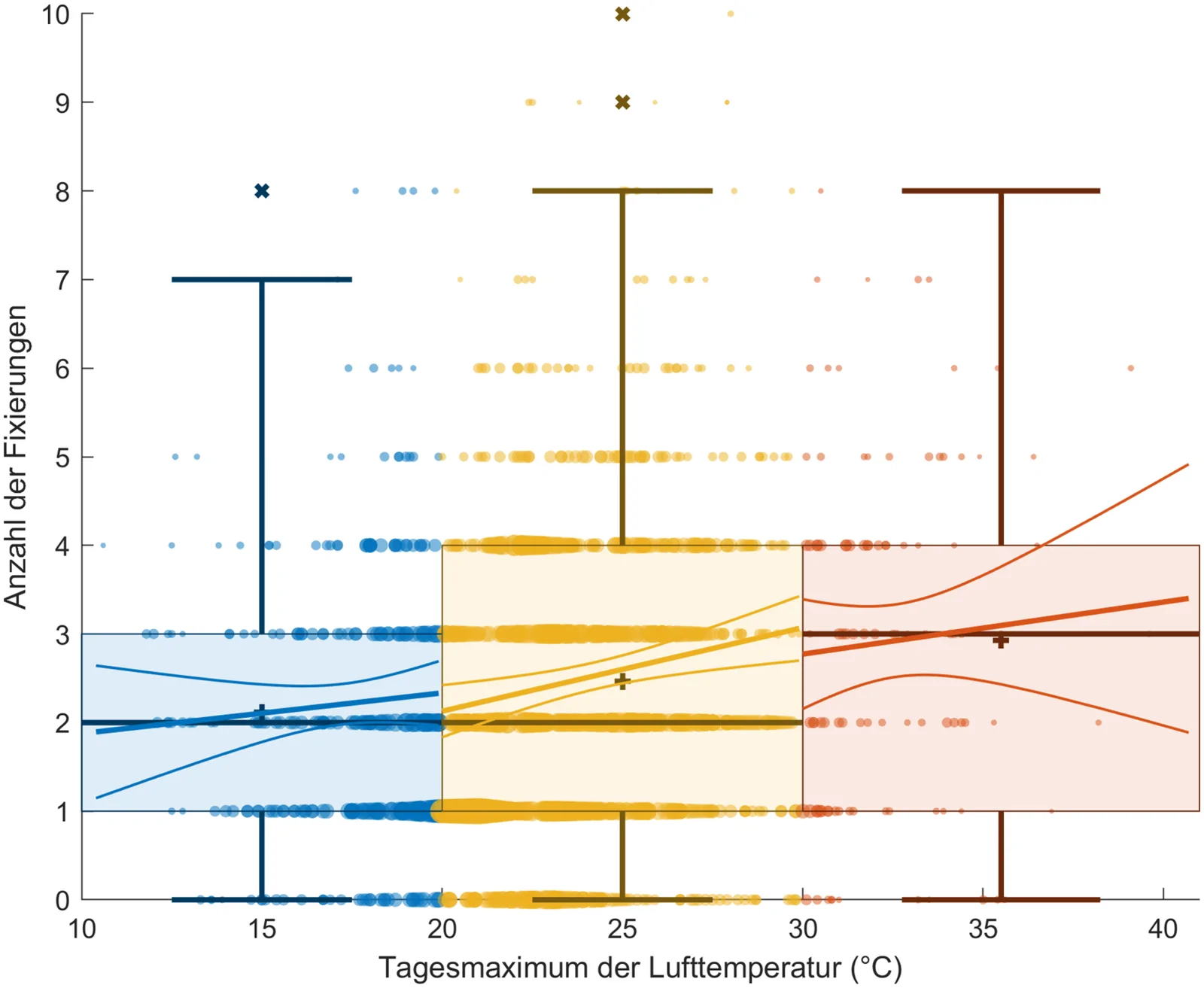
Anzahl der Fixierungen in Abhängigkeit vom Tagesmaximum der Lufttemperatur über die Jahre 2018 bis 2024. Separate Berechnungen für die Temperaturbereiche < 20 °C, 20–30 °C sowie > 30 °C. Die Boxplots zeigen den Mittelwert (+), den Median (*horizontale Linie*), den Interquartilsabstand (*Box*) sowie die Maximal- und Minimalwerte („Whisker“) und Ausreiser (x) separat für die Intervalle < 20 °C (*blau*), 20 bis 30 °C (*gelb*) und > 30 °C (*rot*). Einzelne Datenpunkte sind als Punkte unter den Plots dargestellt. Die Berechnung für den Bereich 20–30 °C ist signifikant (*p* < 0,001, r = 0,128)

Separat gerechnet zeigte sich ebenfalls eine signifikante Korrelation zwischen der Anzahl der Fixierungen sowie dem Tagesmaximum der Lufttemperatur bei Werten zwischen 20–30 °C (*p* < 0,001, r = 0,128). Die separate Berechnung für die Bereiche unter 20 °C sowie über 30 °C zeigte eine Tendenz jedoch keinen signifikanten Zusammenhang (Abb. 1).

## Diskussion

Zusammenfassend konnten wir unsere oben genannte Hypothese von zunehmenden FEZM (Anzahl der Fixierungen) zur Eindämmung aggressiver Verhaltensweisen bei erhöhter Umgebungstemperatur in der stationären psychiatrischen Versorgung bestätigen.

Als theoretisches Model wurde bereits diskutiert, dass heißes Wetter bzw. hohe Umgebungstemperaturen gewalttätiges Verhalten aufgrund von Frustration, Unbehagen, gesteigerter Impulsivität und Aggression begünstigen kann. Eine weitere Theorie diskutiert, dass die Änderung der Umgebungstemperatur das Routineverhalten von Menschen ändert (z. B. andere Aktivitäten im Freien und soziale Kontakte sowie Schlafgewohnheiten) und hierdurch mögliche interpersonelle Konflikte verstärken könnte [6]. Diese Aspekte könnten auch einen Teil der Effekte im Hinblick auf psychiatrische Patient*innen erklären. Bei psychiatrischen Patient*innengruppen zeigen sich je nach Kohorte bereits temperaturunabhängig Raten von 15–20 %, bei denen gewalttätiges Verhalten zu FEZM führt [7]. Hinzu kommen nunmehr Daten, die zeigen, dass erhöhte Umgebungstemperaturen aggressive Verhaltensweisen weiter verstärken können [2] und in einer amerikanischen Untersuchung signifikant mit einer Zunahme notfallmäßiger Vorstellungen psychiatrischer Patient*innen in einer Notaufnahme assoziiert waren [3].

Durch die hier erfolgte Datenerhebung zeigt sich ein signifikanter Effekt bezüglich des Tagesmaximums der Lufttemperatur in Sommermonaten auf die Notwendigkeit zu FEZM in der stationären psychiatrischen Versorgung. Diese Effekte stehen im Einklang mit Daten aus Süddeutschland aus früheren Jahren (2007–2019). Die Zahl der aggressiven Vorfälle war hier an „Hitzetagen“ (≥ 30 °C) signifikant höher und es zeigte sich eine Dosis-Wirkungs-Beziehung zwischen der steigenden Temperatur und der Anzahl aggressiver Vorfälle, jedoch nicht für die Zwangsmaßnahmen [2].

Wir können nun Daten für Westdeutschland in Bezug auf FEZM sowie einen Effekt durch höheres Lebensalter, männliches Geschlecht und im Falle der Fixierungen durch das Vorliegen einer spezifischen Diagnose aus dem Bereich des schizophrenen Formenkreises berichten. Interessanterweise zeigte eine große US-Studie keinen Zusammenhang zwischen dem Patient*innenalter und der Anzahl an Patient*innenkontakten in der Notaufnahme während Hitzetagen auf, aber wie bei uns Zusammenhänge für das männliche Geschlecht sowie für Regionen, die weniger an die Hitze gewöhnt sind [3]. Gewalttätiges und aggressives Verhalten tritt nach Studienlage eher bei Männern auf und ist bedingt durch ein komplexes Zusammenspiel neurobiologischer und psychosozialer Faktoren [8]. Zusätzlich gibt es Hinweise aus Québec, Kanada, die zeigen, dass auch drei Tage nach einer Hitzeperiode die Aufnahmerate von an Schizophrenie erkrankten Patient*innen deutlich erhöht ist [9]. Die Kumulation von Patient*innen im stationären Setting kann bekannterweise zu dem Phänomen des „overcrowding“ führen, was in der Folge zu einer weiteren Verstärkung aggressiver Verhaltensweisen und vermehrten Fixierungen, aber auch zu vermehrtem Stresserleben des Personals beitragen könnte.

Bei der Betrachtung des genannten Zusammenhanges muss auch an die Interaktion zwischen Patient*innen und dem therapeutischen Personal gedacht werden, da sich die erhöhten Temperaturen auf beide Gruppen auswirken können. Hinsichtlich der Interaktion von Patient*innen und Teammitgliedern ist gut bekannt, dass Faktoren wie Geschlecht und Alter, aber auch der Bildungsstand die Wahrnehmung aggressiver Verhaltensweisen beeinflussen [10]. Interessanterweise zeigte sich neben der Korrelation zwischen dem Tagesmaximum der Lufttemperatur und der Anzahl der Fixierungen, dass auch die Anzahl der Fixierungen korrelierend mit dem Tagesmaximum der Lufttemperatur bereits isoliert betrachtet für den Bereich zwischen 20–30 °C signifikant ist, sodass auch schon in diesem Bereich eine Sensibilisierung für FEZM diskutiert werden sollte. Für die Zukunft wäre zu diskutieren, ob es bei noch höheren Temperaturbereichen (z. B. um 40 °C) zu einem weiteren Anstieg oder auch wieder einer Abnahme des Verhaltens, welches zu FEZM führt, kommt. Da für diese Bereiche jedoch nur wenige Messpunkte vorliegen, ist dies schwer zu beurteilen. Für die Untersuchung des Zusammenhangs des Tagesmaximums der Lufttemperatur und Fixierungen für Bereiche unter 20 °C Außentemperatur muss wiederum vermerkt werden, dass in den Klinikgebäuden Heizkörper vorhanden sind (Abb. 1). Klimaanlagen waren auf unseren Stationen nicht vorhanden. Temperaturmessungen auf unseren Stationen lagen im genannten Zeitraum nur sporadisch vor und gaben richtungsweisend an, dass beispielsweise bei einer Außentemperatur von 30 °C eine Innentemperatur auf einer der Stationen von um die 25 °C gemessen wurde.

## Limitationen

Es ist anzumerken, dass die verwendeten Tagesmaxima der Lufttemperatur nicht zur gleichen Zeit, wie der in der Klinik dokumentierte Vorfall gemessen wurden und auch nicht am gleichen Ort, sodass hiervon abweichende Werte während des Vorfalls wahrscheinlich sind. Zukünftige Studien sollten also genaue Vor-Ort-Messungen auf den Stationen vornehmen. Klimaanlagen auf den Stationen könnten einen gegenregulatorischen Effekt haben. Es handelt sich um eine monozentrische Auswertung und ein aggressives Verhalten wurde zudem nicht systematisch auf den Stationen erfasst.

## Fazit für die Praxis

Eine Sensibilisierung für den Zusammenhang von Hitze und Zwangsmaßnahmen ist gerade im akutpsychiatrischen Bereich besonders wichtig, könnte aber sicherlich auch für andere Bereiche des Gesundheitswesens sinnvoll sein. Trainingskonzepte für das Personal könnten hier hilfreich sein. Eine effektive Klimatisierung psychiatrischer (und anderer) Krankenhausbereiche sowie die Einführung und Umsetzung geeigneter Hitzeschutzkonzepte scheint gerade auch in psychiatrischen Kliniken besonders wichtig zu sein.
